# Trail Making Test Performance Using a Touch-Sensitive Tablet: Behavioral Kinematics and Electroencephalography

**DOI:** 10.3389/fnhum.2021.663463

**Published:** 2021-07-01

**Authors:** Zhongmin Lin, Fred Tam, Nathan W. Churchill, Fa-Hsuan Lin, Bradley J. MacIntosh, Tom A. Schweizer, Simon J. Graham

**Affiliations:** ^1^Department of Medical Biophysics, Faculty of Medicine, University of Toronto, Toronto, ON, Canada; ^2^Physical Sciences, Sunnybrook Research Institute, Toronto, ON, Canada; ^3^Keenan Research Centre for Biomedical Science, St. Michael’s Hospital, Toronto, ON, Canada; ^4^Division of Neurosurgery, St. Michael’s Hospital, Toronto, ON, Canada

**Keywords:** time-frequency analysis, partial least squares, neuropsychological tests, computerized tablet, Trail Making Test, EEG

## Abstract

The Trail Making Test (TMT) is widely used to probe brain function and is performed with pen and paper, involving Parts A (linking numbers) and B (alternating between linking numbers and letters). The relationship between TMT performance and the underlying brain activity remains to be characterized in detail. Accordingly, sixteen healthy young adults performed the TMT using a touch-sensitive tablet to capture enhanced performance metrics, such as the speed of linking movements, during simultaneous electroencephalography (EEG). Linking and non-linking periods were derived as estimates of the time spent executing and preparing movements, respectively. The seconds per link (SPL) was also used to quantify TMT performance. A strong effect of TMT Part A and B was observed on the SPL value as expected (Part B showing increased SPL value); whereas the EEG results indicated robust effects of linking and non-linking periods in multiple frequency bands, and effects consistent with the underlying cognitive demands of the test.

## Introduction

The Trail Making Test (TMT) is widely used in behavioral neuroscience and in the clinic as part of neuropsychological test (NPT) batteries, to assess frontal lobe function and to assist in diagnosis of brain disease (Halstead, 1947; Lezak et al., 2012). This pen-and-paper test assesses cognitive processes such as visual search, visual planning, visuomotor control, as well as attention and memory (Halstead, 1947; Reitan, 1971; Stuss et al., 2001; Strauss et al., 2006; Lezak et al., 2012). There are two parts (A and B), each that involve linking a total of 25 randomly placed items in ascending order. Part A (TMT-A) involves linking numbers (1-2-3-4-5…) and Part B (TMT-B), which is more challenging, involves linking numbers alternating with letters (1-A-2-B-3-C…). Standardized protocols require the test recipient to perform the TMT as fast as possible without lifting the pen from the paper (Corrigan and Hinkeldey, 1987; Gaudino et al., 1995; Bowie and Harvey, 2006; Strauss et al., 2006; Lezak et al., 2012). Each part is typically scored by directly measuring the completion time and recording the number of errors; whereas derived scores of the completion times, such as the difference (B-A) and the ratio (B/A) have also been employed to de-emphasize the visuo-motor aspects of performance and to emphasize the cognitive aspects (Corrigan and Hinkeldey, 1987; Stuss et al., 2001; Bowie and Harvey, 2006; Muir et al., 2015).

Despite wide use of the TMT, the underlying brain activity that supports the performance of this test is not understood in detail. Early TMT studies focused on behavioral measures as an indirect indicator of brain state and cognition. Through correlations in behavioral measures with other NPTs, the neuropsychological correlates of the TMT have been demonstrated including visuospatial abilities (Larrabee and Curtiss, 1995; Robins Wahlin et al., 1996), set-switching (Arbuthnott and Frank, 2000; Kortte et al., 2002), and working memory (Sánchez-Cubillo et al., 2009), establishing test validity and sensitivity to brain damage. By examining the behavioral deficits associated with TMT performance in patients with brain lesions, key neuroanatomical correlates such as the left dorsolateral prefrontal cortex (Stuss et al., 2001; Yochim et al., 2007; Barbey et al., 2012; Miskin et al., 2016) and anterior cingulate cortex (Gläscher et al., 2012) have also been identified.

Non-invasive tools for measuring brain activity, such as functional magnetic resonance imaging (fMRI), are starting to provide new opportunities for deeper, more comprehensive investigation of the neuroanatomical and neurophysiological correlates of the TMT (and other NPTs). However, it is challenging to adapt pen-and-paper NPTs so that they can be administered in an MRI system while maintaining ecological validity (i.e., with behavioral performance that generalizes to real-world settings). To study the brain activity associated with TMT performance in healthy adults using fMRI, researchers initially developed several different strategies such as a verbal version of the TMT using covert speech (Moll et al., 2002), a motor TMT using a fiber optic-based drawing device (Zakzanis et al., 2005), a visual TMT involving covert linking responses (Allen et al., 2011), and a computerized TMT using button press responses (Jacobson et al., 2011). Although all four studies confirmed the involvement of the dorsolateral prefrontal cortex, only the former two identified the anterior cingulate cortex. The evidence from these studies is not definitive, but it strongly suggests that simplification of visual and motor components of the TMT failed to be fully representative of the naturalistic writing and drawing performance in the real test – and of the associated brain activity.

To address this limitation, fMRI-compatible tablet technology was developed to approximate naturalistic TMT responses during fMRI (Tam et al., 2011). Using a resistive touchscreen and a stylus with force sensor, the first tablet prototype provided visual feedback of tablet interactions with excellent utility. However, the test recipient was unable to see their hand grasping and manipulating the stylus while interacting with the tablet, which was thought to impact behavioral performance under some circumstances (Karimpoor et al., 2017). A second-generation prototype was developed to address this problem by providing visual feedback of hand position (VFHP), enabling the participant to view the display of test stimuli overlaid with live video of hand/stylus/touch-surface interactions in an augmented reality environment (Karimpoor et al., 2015). This approach provides increased awareness of the hand and stylus position in real time, and thus enables test recipients to perform tablet interactions and undertake the TMT with enhanced ecological validity. Furthermore, as the tablet interactions are digitized and recorded on a computer, kinematic metrics can be developed for a much more nuanced characterization of TMT performance than is achievable with the standard scoring procedures.

Recent fMRI research has employed the second-generation tablet prototype to study the brain activity of TMT performance in different populations of healthy adults. Studying young healthy adults, Karimpoor et al. (2017) identified bilateral brain activations in regions associated with somatosensory and motor processes, visual perception, imagined movement and visual search in TMT-A versus Control (visual fixation) and TMT-B versus Control contrasts (Richter et al., 2000; Nobre et al., 2003). Left lateralized brain activations in regions associated with executive function, motor planning, visual search and performance monitoring were found in the TMT-B versus TMT-A contrast with VFHP (Moll et al., 2002; Nobre et al., 2003; Brown and Braver, 2005; Zakzanis et al., 2005; Allen et al., 2011; Lezak et al., 2012), including the dorsolateral prefrontal cortex and anterior cingulate cortex, consistent with previous findings (Stuss et al., 2001; Gläscher et al., 2012). More recently, Talwar et al. (2020) demonstrated age-related decrements in behavior and brain activation during TMT performance. Compared to younger adults, older adults exhibited poorer task performance and reduced TMT-related brain activity in the bilateral occipital, temporal, and parietal lobes, consistent with previous reports of age effects in TMT behavior (Giovagnoli et al., 1996; Robins Wahlin et al., 1996; Drane et al., 2002; Tombaugh, 2004; Talwar et al., 2020).

Functional MRI signals provide a detailed picture of brain activity at millimeter spatial resolution, but typically with low temporal resolution (approximately seconds) because the signals arise from sluggish neurovascular coupling effects. Alternatively, electroencephalography (EEG) uses scalp electrodes to record voltage fluctuations from the ionic currents produced by neural activity, with high temporal resolution (approximately milliseconds). The EEG method thus holds promise for revealing the dynamic mental processes involved in TMT performance. The spatial resolution for localizing neural activity with EEG is typically much lower (centimeters) than that achievable by fMRI, and is primarily limited to regions immediately below the scalp. Nevertheless, it should be possible to use EEG recordings to fill a gap in knowledge about the time and frequency features of neural activity responsible for TMT performance, and to determine whether the EEG signals are distributed among the electrodes in a manner that is consistent with fMRI results.

One EEG study of TMT performance was conducted approximately 35 years ago. Signals were recorded from only four electrodes that divided the skull surface into quadrants (Lorig et al., 1986). Both TMT parts were found to generate high frequency (14-30 Hz) oscillatory activity, with left lateralized posterior brain activity more evident in TMT-B (Lorig et al., 1986), consistent with subsequent TMT studies (Stuss et al., 2001; Karimpoor et al., 2017; Talwar et al., 2020). With modern EEG recording and data analysis technology, as well as better understanding of TMT-related brain activity, the primary goal of the present study was to examine in young healthy adults the differences in the temporal aspects of behavioral performance and in the EEG signal content between TMT-A and TMT-B. As part of this goal and using methodology consistent with recent tablet-based fMRI studies (e.g., Talwar et al., 2020), tablet technology incorporating VFHP (Karimpoor et al., 2015) was used to provide quantitative kinematic recording of TMT performance to inform the interpretation of EEG signals. Regarding behavioral performance, it was hypothesized that the behavioral metrics are significantly different across TMT parts, consistent with past TMT literature. With respect to EEG, it was hypothesized that the spatial patterns of electrode activation are significantly different (1) across TMT parts, consistent with previous functional neuroimaging literature investigating a similar study population; and (2) across time periods with significantly different aspects of performance during each TMT part, such as visual search and linking behavior, as quantified from tablet-based kinematic metrics. These initial results permit preliminary qualitative discussion of the pertinent fMRI and EEG literature relating to TMT performance, and set the stage for future simultaneous EEG-fMRI studies.

## Materials and Methods

### Participants

The study was approved by the Research Ethics Board of Sunnybrook Health Science Centre in Toronto. All research was performed with the free and informed consent of the participants, who were right-handed based on self-report and behavioral monitoring, were English-speaking, were free from EEG exclusion criteria (e.g., discomfort with gel on the scalp), and were free from past or present neurological and psychiatric impairments. Participants were recruited from graduate students at the University of Toronto and research personnel at Sunnybrook Research Institute in Toronto.

Sixteen healthy young adults participated in the study (8 male, 8 female, age range: 19-27, mean age 21.3 ± 2.8 years). All participants performed the tablet-based TMT with EEG in an acoustically shielded room, supervised by an experienced test administrator (ZL).

### Trail Making Test Design

**FIGURE 1:**
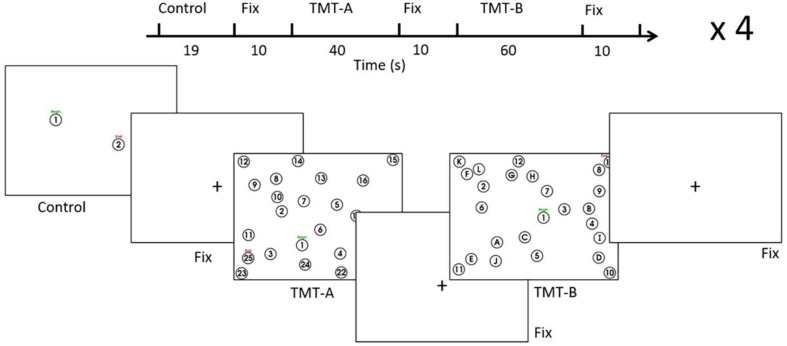
Task design for the EEG experiment. See text for details. Fix = fixation, TMT = Trail Making Test.

The TMT was administered according to a format (Figure 1) commonly adopted in the literature (Reitan, 1971; Corrigan and Hinkeldey, 1987; Gaudino et al., 1995; Lezak et al., 2012) using a stimulus/response computer (Intel i5-2500 4-core CPU, 16 GB RAM). The TMT-A involved linking encircled number stimuli from 1 to 25, which were distributed pseudo-randomly across the screen. The distribution was based on the standard TMT with a 90° rotation to fit the “landscape” format of the display. The TMT-B involved linking number stimuli (1-13) alternating with letter stimuli (A-L) in another pseudo-random spatial distribution. Based on the standardized and previous TMT instructions used in the field (Corrigan and Hinkeldey, 1987; Gaudino et al., 1995; Bowie and Harvey, 2006; Strauss et al., 2006; Lezak et al., 2012; Talwar et al., 2020), participants were asked to connect the circles from “Begin” to “End” as fast and as accurately as possible, without lifting the stylus from the tablet. Prior to the EEG experiment, the TMT performance was also demonstrated to participants using test samples of TMT-A and TMT-B, as a supplement to oral instructions. Similar to previous fMRI studies of the TMT that adopted a block design (Karimpoor et al., 2017; Talwar et al., 2020), the design (one run) contained four trials of control tasks (8 repeats, approximately 19 s), TMT-A (40 s), and TMT-B (60 s), separated by visual fixation (10 s). The control tasks involved linking two items from “Begin” to “End” (1-2). Starting with one TMT-A and one TMT-B version derived from the standard TMT arrangement, the variants for each trial were created by either rotating the stimulus distribution by 180° or swapping between number-only stimuli and number-letter stimuli, or both. This procedure was undertaken to minimize the contribution of different stimulus distributions to the performance difference between TMT-A and TMT-B (Gaudino et al., 1995). The visual fixation task served as the baseline condition in the present study, in which participants focused on a black crosshair located centrally on a white display. Each participant underwent two runs of the task design, completing a total of 8 trials for TMT-A and TMT-B, respectively. The task design was implemented and administered by a custom program written using E-Prime Software (version 2.0.10.356, Psychology Software Tools, Sharpsburg, PA, United States) on the stimulus/response computer, which received and interpreted the stylus position recorded on the tablet, providing task-related performance feedback as ink marks superimposed on the task stimuli. During EEG recording, tablet behavioral performance was recorded as time-varying (x, y) coordinates indicating stylus position on the tablet. The coordinates were sampled at a rate of approximately 40 Hz in E-Prime and logged into a computer text file upon trial completion.

### Tablet Technology

The present study incorporated the same tablet technology used in a number of fMRI studies of tablet-based TMT performance (Tam et al., 2011; Karimpoor et al., 2017; Talwar et al., 2020), facilitating comparison with the literature. The digitizing tablet is one of the few devices of its kind that permits simultaneous, combined fMRI-EEG experiments in the future. Concurrently employing the tablet and EEG not only enables direct comparison with previous tablet TMT results, but also enables preliminary investigation of the relationship between tablet-based TMT performance and EEG recordings, as a necessary preliminary step toward future multimodal experimental design and data analysis. The tablet contained a patented resistive transparent touchscreen (Microtouch, Model #RES-6.4-PL4, 3M, St. Paul, MN; 16 cm diagonal; 13 cm × 10 cm active area) along with its matching controller board (Microtouch, Model #SC400, 3M, St. Paul, MN, United States). Connected to the stimulus/response computer as an input device, the tablet system detected stylus contact force and recorded stylus coordinates, for subsequent display as ink marks. The stylus tip was also equipped with a force sensor (FSR 400, 30-49649, Interlink Electronics, Carmarillo, CA, United States) to detect the presence and magnitude of the exerted force. However, the contact force was not part of the scope of the study and thus the presence of the force was logged, rather than the magnitude.

**FIGURE 2:**
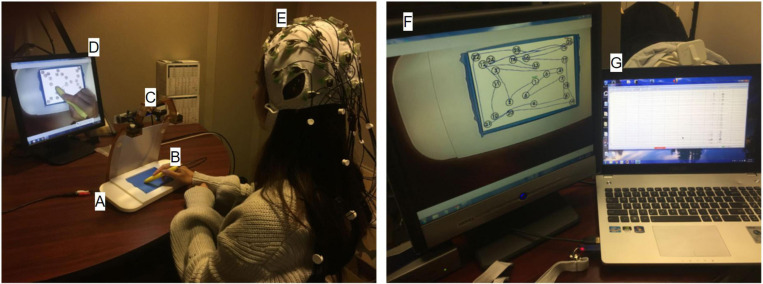
The experimental setup inside (left) and outside (right) the acoustically shielded room. **(A)** Touch-sensitive tablet, **(B)** Stylus, **(C)** Video camera, **(D)** Visual feedback of stimulus response and hand position, **(E)** EEG cap and electrodes, **(F)** Stimulus/response computer, **(G)** EEG recording computer.

As shown in Figure 2, the tablet technology and stimulus/response computer were configured for EEG experiments in an acoustically shielded room (Industrial Acoustics Company, The Bronx, NY, United States). The tablet with stylus was placed on the desk in front of the participant to ensure a naturalistic and comfortable writing posture. In addition to the task-related stimuli captured by the stimulus/response computer, the tablet was mounted with a color video camera (12M-i with 4.3 mm lens, MRC Instruments GmbH, DEU) and a battery-powered illuminator to enable VFHP. The touchscreen was calibrated on the stimulus/response computer to ensure the spatial accuracy of the task-related stimuli. Enabled by drivers and software on an additional video processing computer (Intel i5-3570 4-core CPU, 8 GB RAM) an interactive augmented reality environment consisting of (a) segmented video of hand/stylus interactions on the touch-sensitive surface of the tablet; (b) the task-related visual stimuli; and (c) the graphical representation of the interactions as ink marks, was displayed to the participant via a computer monitor (LG L1718S, 17-inch diagonal, 1280 × 1024 resolution, 60 Hz refresh rate). The monitor was positioned approximately 65 cm in front of the participant, creating a subtended visual angle of approximately 16°. The surface of the tablet was covered in blue tape, enabling a video “mask” to be created of the hand and stylus only (zero signal intensity elsewhere) by segmenting the acquired video of task-related performance based on color content. The video camera was angled appropriately to ensure that the blue taped touchscreen was fully captured for seamless superimposition of task-related visual feedback.

### Electroencephalography Recording

The EEG data were recorded with a sampling rate of 5000 Hz using an actiCAP active electrode system (32 channel), actiCHamp amplifier system, and BrainVision Recorder software (version 1.21.0402, Brain Products GmbH, Gilching, DEU) on a laptop computer (Intel i7-3610QM 4-core CPU, 8 GB RAM). The EEG amplifier collected and amplified electrophysiological signals recorded by scalp electrodes and transmitted them to the recording laptop over a USB (universal serial bus) cable. The stimulus/response computer was also programmed using E-Prime to send parallel port triggers marking the onset of each task phase (control, fixation, TMT-A, TMT-B), to a dedicated trigger port on the EEG amplifier. The head circumference was measured for each participant and the 32 electrodes along with a ground electrode were mounted on the electrode cap of appropriate head size (EASYCAP GmbH, Herrsching, DEU). The participant was then outfitted with the cap, which was adjusted by the experimenter (Z.L.) to ensure that electrode Cz was at the topographic center of the head. Conductive electrolyte gel (SuperVisc 1000 gr.; EASYCAP GmbH, Herrsching, DEU) was then injected (using a blunt needle) through the electrode aperture between the individual electrode and the scalp to minimize the electrical impedance to less than 25 kΩ. The impedance was actively monitored during the gel injection, but the values were not recorded.

### Tablet Data Analysis

As an initial effort to explore the utility of tablet TMT in EEG, the present data processing approaches are still prototypical. Given the highly variable behavioral patterns across participants, video inspection provides a reasonable starting point to evaluate conventional TMT performance (i.e., completion time, number of errors) versus an automated analysis with predetermined parameters. Using a custom MATLAB program (The MathWorks, Inc., Natick, MA, United States), the TMT performance in each individual trial was rendered as a video file of tablet ink marks superimposed on the test stimuli. The video files were then carefully visually inspected to quantify the completion times, number of errors, correct links, and total number of links. Linking errors were identified based on the sequence of linked items. For example, in TMT-A, 2-3 is the only correct link that starts with 2, whereas 2-1, 2-4 and others are error links. The intentional target item of a link was judged based on the pause and turn at the item. For example, if link 2-3 crossed 6 in the link path with no noticeable pause and turn at 6, then no errors were logged and 2-3 was counted as a correct link. Descriptive statistics were computed for the completion times, number of errors, correct links, and total links in TMT-A and TMT-B separately. Wilcoxon signed rank tests were also used to evaluate the effect of TMT part in these metrics.

Inspired by digitized metrics in other neuropsychological tests such as the clock-drawing test (Werner et al., 2006; Heinik et al., 2010), the seconds per link (SPL) was adopted from past research to evaluate TMT performance over the entire test trial (Karimpoor et al., 2017). The metric was originally developed to accommodate the fixed block duration typical of many task-based fMRI experiments, where the participant might not complete the TMT trial within the specified time. As an extension from the conventional metrics, the SPL eliminated the possibility of a “ceiling effect” in completion time. The SPL of each TMT trial was calculated by dividing the completion time (either the block duration, or the time required to complete all links) by the number of correct links. (Notably, the numerator in this calculation includes both correct as well as incorrect links, such that information about linking errors is included in the SPL metric. However, this has negligible implications for TMT investigations of young healthy adults, who perform the test with minimal errors – as confirmed in the Results.) Speed timeseries obtained from the digitizing tablet also served as an important metric to conduct offline analysis on within-test behavioral dynamics (see description later below).

Several initial analyses were conducted to direct the course of subsequent statistical testing and to ensure data consistency. Regarding the latter issue, given that EEG signals are of low amplitude and contaminated by substantial electronic noise, it is essential to improve signal detection power by performing analysis over repeated TMT trials (see section “Trail Making Test Design” for methodological details). There is a similar imperative in fMRI experiments, hence the adoption of a block design protocol in the present study based on TMT-related fMRI literature (Karimpoor et al., 2017). Prior to including all trials in the analysis, however, it is first necessary to evaluate whether effects such as habituation or learning introduce systematic biases. Motivated by similar considerations and testing in a recent fMRI study (Talwar et al., 2020), the following procedure was adopted. First, the overall SPL distributions in TMT-A and TMT-B were visualized using histograms and each was assessed for normality using the one-sample Kolmogorov-Smirnov test. As both SPL distributions were significantly different from a normal distribution (TMT-A: *p* < 0.001, TMT-B: *p* < 0.001), subsequent SPL analyses employed non-parametric statistical tests. Kruskal-Wallis tests were conducted on SPLs in TMT-A and TMT-B separately to assess the effect of trials, which revealed significant effects (TMT-A: *p* < 0.01, TMT-B: *p* < 0.001). Using Dunn & Sidák’s Approach to perform a *post hoc* multiple pairwise comparison of the trial means, the SPL value of Trial 1 in TMT-A was found to be significantly larger than that of Trials 5-8 (Trial 1-5: *p* < 0.001; Trial 1-6: *p* < 0.01; Trial 1-7: *p* < 0.05; Trial 1-8: *p* < 0.05). No other significant differences in SPL value were detected among the remaining trials for TMT-A. For TMT-B, a similar effect was observed (Trial 1-5: *p* < 0.001; Trial 1-6: *p* < 0.05; Trial 1-7: *p* < 0.01; Trial 1-8: *p* < 0.05). Thus, to report EEG results from as many trials of self-consistent data as possible, without systematic trends due to factors such as habituation and learning, the behavioral and EEG recordings of Trial 1 in both TMT parts were excluded from all subsequent analyses. After these exclusions, the effect of trial was absent across the remaining seven trials for both TMT parts, permitting the data to be pooled to enhance the power to detect EEG effects. The overall SPL distributions were then compared across participants using the two-sample Kolmogorov-Smirnov test. In addition, as part of visually inspecting these distributions, bootstrapping was used to estimate 95% confidence intervals of the mean value at each sampling bin. The skewness of the individual distributions was computed for subsequent Wilcoxon signed rank tests between TMT-A and TMT-B. Lastly, the SPLs were averaged across the seven trials for each participant for both TMT parts, and another Wilcoxon signed rank test was performed to assess the effect of TMT part on the SPL averages.

Additional kinematic analysis was conducted to characterize tablet interactions during TMT performance. Specifically, the time derivative of stylus position coordinates (x, y) was estimated using finite differences divided by the sample period, resulting in plots of speed (in pixels/second, px/s) versus time for each participant for both TMT parts. This method of estimating derivatives is prone to spikes or outliers and other noise; thus the speed plots were further processed using a custom Sigma filter for spike removal (Solomon et al., 2001), and a low pass filter with 5 Hz cut-off frequency. The Sigma filter was a sliding time window operator (window length = 151 ms) that provided smoothing by substituting outlier values outside the range of two standard deviations with the average of adjacent non-outlier values. The 5 Hz cut-off frequency for the low pass filter was chosen by observing that 95% of the spectral content of speed time-courses was below 5 Hz. The speed data were then interpolated using cubic splines and resampled at a rate of 1000 Hz, to match the down-sampled EEG data used in the subsequent analysis (see section “EEG Data Analysis” below).

**FIGURE 3:**
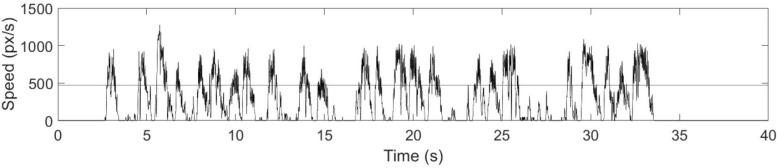
Speed time course of the stylus during TMT-A performance, for a representative participant. Linking periods are characterized by rapid acceleration to peak stylus speed, followed by similar deceleration, whereas non-linking periods are characterized by much lower stylus speed. The horizontal line indicates the threshold separating linking and non-linking periods.

The speed time-courses primarily showed two characteristic behavioral features (Figure 3): (1) periods of slow stylus speed, presumably due to preoccupation with visual search activities and subsequently called “non-linking periods;” and (2) periods of acceleration to peak speed, followed by similar deceleration, characteristic of purposeful movements to link stimuli and subsequently called “linking periods.” Due to the absence of other EEG and behavioral time markers to identify the onsets and offsets of these periods, the linking and non-linking periods were separated using a speed amplitude threshold. The speed threshold was established manually by slowly increasing the candidate speed threshold from 0 px/s with a constant step for each participant until the linking periods were readily identifiable (i.e., showing a consistent pattern of rapid acceleration and deceleration across individual links), and the number of linking periods matched the total number of links previously determined by visual inspection of the video recordings. As a result, the speed threshold varied across each trial, depending on the peak speed of the fastest non-linking period. Varying speed threshold is a preliminary approach to determine linking and non-linking periods offline, solely from the speed timeseries. Subsequently, time indices specifying linking and non-linking periods were obtained for every trial, enabling the average speed of individual links, non-linking periods, and linking periods to be calculated within-test for each participant and for both TMT parts. Average link speed refers to the average stylus speed over the time course of performing an individual link. In other words, every single link has its own average speed within a given TMT trial. Similar to SPL, the distributions of average link speed, non-linking period and linking period were quantified and visualized using histograms, with 95% confidence intervals estimated as described above. The overall distributions across participants were compared between TMT parts via two-sample Kolmogorov-Smirnov tests. The distributions for individual participants were also examined to compute the 95% confidence intervals for the mean values represented in the overall distributions, and the skewness for Wilcoxon signed rank tests between TMT parts. In addition, the average link speed, non-linking period, and linking period were averaged across seven trials for both TMT parts with the first trials excluded. Wilcoxon signed rank tests were subsequently performed to assess the effect of TMT part on these averages.

### Electroencephalography Data Analysis

The EEG data were analyzed in three steps, as outlined below. First, pre-processing was undertaken to obtain data segments of interest with improved quality. Second, time-frequency analysis was performed using complex Morlet wavelet convolution to extract spectral information while maintaining temporal resolution. Third and finally, average time-frequency powers were computed to assess effects of TMT part and time period using task-based partial least square (PLS) regressions.

Using EEGLAB (Delorme and Makeig, 2004) and custom MATLAB software, the EEG data were down-sampled to 1000 Hz, passed through a band-pass finite impulse response filter with 0.1-100 Hz bandwidth, and decomposed using independent component analysis (ICA) to remove spurious eye blinks, eye movements, muscle noise and channel noise. Identification of artifactual ICs was conducted using an automated classifier algorithm based on machine learning (Pion-Tonachini et al., 2019a), which was trained using large scale EEG data and expert labels to reliably and accurately label the signal content of an IC (i.e., brain, artifact, and noise) as percentages (Pion-Tonachini et al., 2019b). The labeled ICs with more than 90% artifact or noise were excluded from further analysis. To maximize the effects of interest in the frontal electrodes, the EEG data were re-referenced to the mean mastoids using the average signal from electrodes TP9 and TP10, situated close to the earlobes. Because artifacts were removed on all electrodes before the re-reference, the EEG signal quality was not compromised by the re-reference and no artifacts were projected back into the signals. With the first trials removed, the EEG data for each participant were subsequently segmented and then concatenated into time-courses specifically for TMT-A and TMT-B. Each TMT trial was segmented along with a 10 s pre-stimulus baseline period, consisting of visual fixation. Given that the TMT stimulus onset was defined as time 0, each trial segment was from -10 to 42 s for TMT-A, and -10 to 62 s for TMT-B. The extra 2 s beyond the task time limit was included in each case to avoid “edge artifacts” during time-frequency decomposition (see immediately below). The analysis included only the EEG power over the available time duration that participants were performing each TMT part within the block duration (as the completion time was variable, and some participants completed a given TMT part faster than the time allotted).

Using custom MATLAB software based on published methodology (Cohen, 2014), individual trials underwent time-frequency decomposition over the frequency range of 0.1–50 Hz using complex Morlet wavelet convolution. Within the frequency range, wavelets were generated with 20 frequencies that increased exponentially. All wavelets had 6 cycles, irrespective of frequency. Based on their frequencies, the wavelets were then assigned to the five major EEG frequency bands: delta (0.3-4 Hz), theta (4-8 Hz), alpha (8-13 Hz), beta (13-30 Hz), and gamma (30-50 Hz). Four wavelets with frequencies closest to the boundary frequencies of EEG frequency bands (e.g., at 4 Hz and 8 Hz) were assigned to both the lower and higher frequency bands, which slightly expands the frequency band ranges to account for individual variability. The total power at each frequency as a function of time was then baseline-corrected using decibel (dB) normalization. The baseline period selected for the analysis was the 10 s visual fixation before each TMT trial. Only 7 s (−8 to −1 s) of the period was used to avoid edge artifacts produced by wavelet convolution. In the time-frequency total power within the completion time of each trial, average time-frequency total power was computed across predetermined linking and non-linking time indices as well as wavelet frequencies assigned to individual frequency bands. For example, in each trial, average power was extracted for the “linking period delta band,” “non-linking period delta band,” “linking period alpha band,” etc. The average time-frequency power was then averaged across the seven trials for each frequency band, time period, and electrode.

**FIGURE 4:**
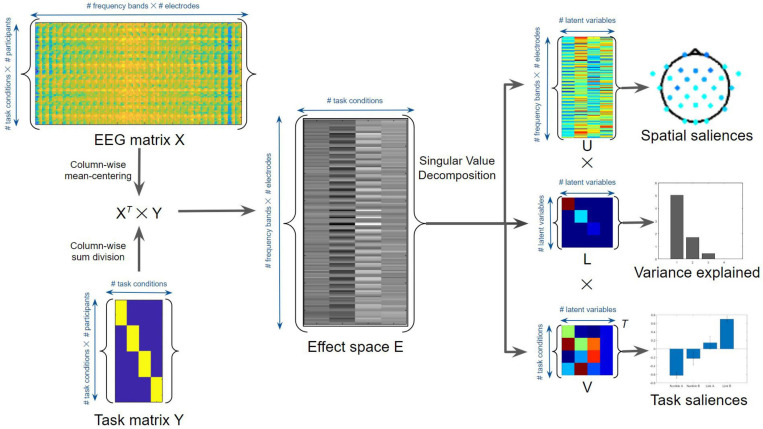
Task Partial Least Squares (PLS) algorithm for the average EEG time-frequency power. See text for details.

To assess and interpret the multivariate task-related effects on EEG time-frequency power using non-parametric statistics, task-based PLS regressions were performed using MATLAB (McIntosh et al., 1996; Lobaugh et al., 2001; Grady et al., 2010; Figure 4). Task PLS was chosen for its ability to identify distributed brain networks from noisy and highly correlated EEG data. The method uncovers the relations between two input matrices (X and Y) that are used to generate a covariance or correlation matrix, by identifying sets of paired latent variables (LVs) derived from X and Y that show maximized covariance. The EEG power data of different task conditions were assigned to the X matrix, whereas the task conditions were considered as the Y matrix or loading vector. The number of potentially significant LVs was set equal to the number of task conditions. To evaluate the main effects of TMT parts and time periods, an omnibus task PLS analysis was first conducted involving the time-frequency power in four task conditions: linking period of TMT-A (Link A), linking period of TMT-B (Link B), non-linking period of TMT-A (Non-link A), and non-linking period of TMT-B (Non-link B). The dimension of the X matrix was thus 64 (16 participants × 4 task conditions) by 150 (30 electrodes × 5 frequency bands), whereas the dimension of the Y matrix was 64 (16 participants × 4 task conditions) by 4 (4 task conditions). The effect space E was derived using the X*^*T*^* × Y cross product matrix, which was then decomposed using singular value decomposition (i.e., E = uLV*^*T*^*, where *T* is the matrix transpose) to generalize eigenvectors and eigenvalues indicating the spatial and task saliences of each latent variable with variance explained. The statistical significance of the LVs was assessed by 1000 permutation resamples. The *p*-Values and bootstrap ratios (BSRs, mean loadings divided by standard deviations) were obtained for all saliences using bootstrapping with 1000 resamples. In addition, the saliences of paired task contrasts were also assessed to compare the strength of contrasts. These saliences were computed by subtracting the salience resamples of one task condition with the permuted salience resamples of another task condition across all combinations of task conditions. The associated mean loadings, standard deviations, and BSRs were then subsequently calculated. Notably, the multiplication between eigenvalues (variance explained) and eigenvectors (spatial and task saliences) gives the portion of the covariance; thus, the saliences are inherently normalized (i.e., unitless) according to the procedure described above.

To further differentiate the spatial pattern for each effect across electrodes, follow-up task PLS subtests were conducted involving paired conditions such as Link B vs. Link A, Non-link B vs. Non-link A, Link A vs. Non-link A, and Link B vs. Non-link B. In addition, the general effect of time period in TMT-A and TMT-B was assessed in a combined comparison by adding the time-frequency power of Link A with Link B, and the analogous computation for the Non-link conditions, permitting a subtest of Link (A + B) vs. Non-link (A + B). The PLS analysis procedures were conducted in analogous fashion to those described above. Non-significant BSRs, as determined after correction for multiple comparisons according to the false discovery rate (FDR) method using *q* = 0.05, were assigned a value of zero for representation in the maps. An additional threshold of |BSR| > 2 was employed to exclude any results that were found to be unstable at a given electrode during the PLS resampling procedure.

## Results

### TMT Performance

Table 1 summarizes behavioral metrics of TMT performance across the participants for the experimental design. The results are averaged over the seven trials and sixteen participants. The Wilcoxon signed rank tests compare the trial averages of TMT-A and TMT-B. Although the mean completion times are smaller than the block duration of the TMT part, there is variability across trials and participants, as some trials were completed within the block duration, and some were not. In addition, the number of errors and correct links indicate that participants performed both TMT parts well. (A participant can complete a maximum of 24 correct links, whereas the number of total links may exceed 24 due to the presence of both correct links and errors).

**TABLE 1:** Behavioral metrics for participants (*n* = 16) performing the tablet TMT*.

	TMT part	Mean	*SD*	Range	*p*-Value
Completion times (s)	A	29.3	6.6	[16.1, 39.9]	*p* < 0.001
	B	37.1	10	[20.5, 59.2]	
Number of errors	A	0.1	0.5	[0, 4]	*p* < 0.05
	B	0.5	1.6	[0, 7]	
Number of total links	A	23.5	1.9	[10, 26]	*p* = 0.2
	B	23.9	0.8	[18, 26]	
Number of correct links	A	23.4	1.9	[10, 24]	*p* = 1
	B	23.4	1.9	[12, 24]	
SPL (s)	A	1.3	0.4	[0.7, 3.9]	*p* < 0.001
	B	1.6	0.6	[0.9, 4.9]	
Speed (px/s)	A	283.8	124.8	[30.2, 661]	*p* = 0.079
	B	273.1	101.4	[67.3, 735.5]	
Linking period (ms)	A	758.8	585.2	[59, 14852]	*p* < 0.001
	B	865.3	582	[112, 17054]	
Non-linking period (ms)	A	563	1026.8	[3, 14795]	*p* < 0.01
	B	723.3	1041.9	[15, 16754]	

*First trials of each TMT part were excluded from the calculation. SD, standard deviation; SPL, seconds per link.

As indicated by the means and signed rank *p*-Values, it is evident that participants exhibited significantly longer completion times, and higher numbers of errors in TMT-B than TMT-A. Smaller mean difference between TMT parts was observed in the number of total links, because both parts have the same stimulus spatial pattern and the same number of links available. As indicated by the means and standard deviations, there was negligible difference in the number of correct links completed for TMT-A and TMT-B, because the vast majority of trials were completed with no errors.

As shown in Table 1, participants exhibited significantly larger values in TMT-B than in TMT-A for SPL (*p* < 0.001), linking period (*p* < 0.001), and non-linking period (*p* < 0.01) but not for average link speed (*p* = 0.079). In addition, the parameter distributions for TMT-A were found to be significantly different than those for TMT-B for SPL (*p* < 0.001), average link speed (*p* < 0.001), non-linking period (*p* < 0.001), and linking period (*p* < 0.001), respectively. In terms of the skewness of the distributions, only average link speed (*p* < 0.01) and non-linking period (*p* < 0.05) demonstrated significant differences between TMT-A and TMT-B.

### Electroencephalography Time-Frequency Power

**FIGURE 5:**
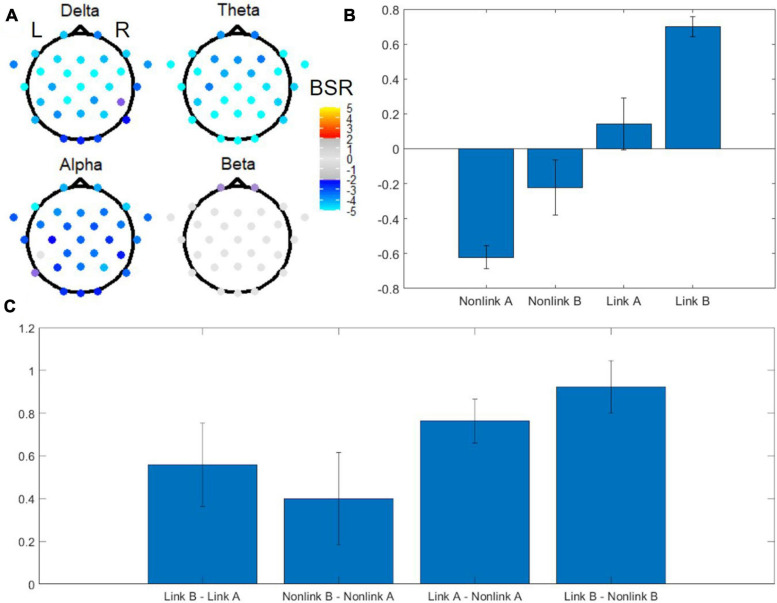
Omnibus task PLS analysis of EEG power for TMT performance. **(A)** BSRs of EEG scalp electrodes in the delta, theta, alpha and beta bands (first latent variable). Significant BSRs are shown according to the color scale given, after correction for multiple comparisons using the false discovery rate (FDR) at *q* = 0.05 and an additional threshold of |BSR| > 2 to remove results that were unstable during the resampling procedure. The spatial pattern for the gamma band is not shown due to lack of statistical significance. **(B)** Mean loadings of task condition weights. Error bars indicate standard deviations. **(C)** Mean loadings of task contrast weights. Error bars indicate standard deviations. L = left, R = right, BSR = bootstrap ratio, Link A = linking period of TMT-A, Link B = linking period of TMT-B, Non-link A = non-linking period of TMT-A, Non-link B = non-linking period of TMT-B.

Digitized tablet metrics have previously been reported in the behavioral neuroscience literature (e.g., Werner et al., 2006); in the present work, they revealed novel performance differences between TMT parts (Table 1), uncovering aspects of within-TMT part performance that are particularly relevant to interpreting the associated EEG signals. The omnibus task PLS with four conditions including linking period of TMT-A, linking period of TMT-B, non-linking period of TMT-A, and non-linking period of TMT-B revealed distinct EEG spatial patterns in each frequency band in the first latent variable, associated with pronounced effects of task (Figure 5). The first latent variable accounted for 64% of the data variance, with no others reaching statistical significance. Electrodes with significant BSRs reflect brain regions that are consistently part of a functional neural network that has the identified task condition loadings across resamples. Among the five frequency bands, delta, theta, and alpha bands exhibited significant bilateral activity patterns across most of the electrodes. Specifically, the delta band showed a widespread spatial pattern with highly negative BSRs around the anterior and central regions, whereas the theta band showed highly negative BSRs in the temporal and occipital lobes as well as central posterior part of the brain. The alpha band showed highly negative BSRs in four frontal electrodes, whereas the beta band only exhibited marginally significant negative BSRs in two frontal electrodes. All BSRs in the gamma band were neither significant after FDR correction nor above the |BSR| > 2 threshold, and thus were not displayed.

Associated with these spatial patterns, the mean loadings of task condition weights exhibited an increasing trend across the tasks arranged in the order (Non-link A, Non-link B, Link A, Link B), with Non-link A showing the strongest negative weight, and Link B showing the strongest positive weight (Figure 5B). An important feature of this weighting pattern was the different influence of time periods and TMT parts to the variance in EEG power (Figure 5C). Specifically, the Link B vs. Non-link B and the Link A vs. Non-link A contrasts were the strongest contributors, followed by Link B vs. Link A, whereas Non-link B vs. Non-link A was the weakest.

**FIGURE 6:**
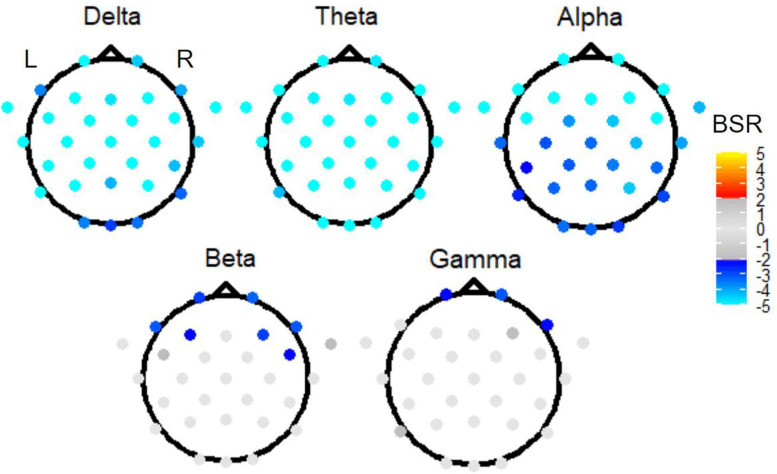
The BSRs of the first latent variable in the Link (A + B) vs. Non-link (A + B) task PLS subtest. All electrode BSRs were FDR-corrected and thresholded as described in the text.

The omnibus PLS results of Figure 5 motivated three follow-up reduced analyses, which are presented in ranked order from largest effect to smallest effect. First, to investigate the effect of time period, task PLS analysis of the Link vs. Non-link periods in both TMT parts revealed widespread BSR spatial patterns after FDR correction and thresholding. The subtask of Link (A + B) vs. Non-link (A + B) is shown in Figure 6 for conciseness. Constrained to fixed values by the task PLS algorithm, the significance (*p*-Value) and explained variance of latent variables are not meaningful in a two task PLS analysis, thus only the first latent variable was reported. Similarly, the mean loadings are not meaningful in this scenario and thus were not reported. Highly negative electrode BSRs were detected across the scalp in the delta band, with exceptions mostly in the occipital regions. The theta band consistently demonstrated highly negative BSRs across the scalp, whereas the alpha band exhibited highly negative BSRs in the bilateral frontal regions. Beta and gamma bands revealed moderately negative BSRs localized in the bilateral frontal regions with a slightly right-lateralized spatial pattern.

**FIGURE 7:**
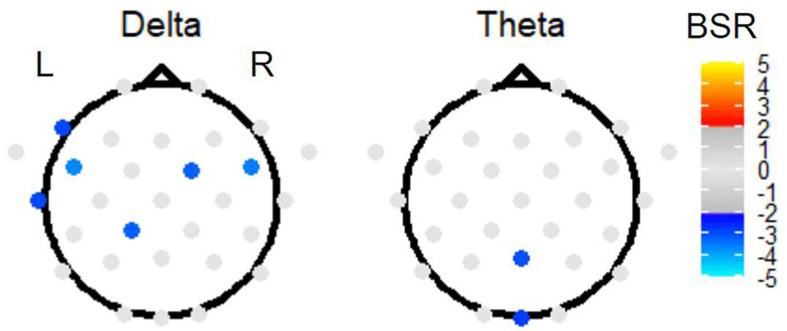
The BSRs of the first latent variable in the Link B vs. Link A task PLS subtest. All electrode BSRs were FDR-corrected and thresholded as described in the text. The electrode spatial patterns in alpha, beta, and gamma bands were not significant.

Second, investigating the effect of TMT parts on EEG power, PLS sub-analysis of only the Link B and Link A data (thus investigating the Link B vs. Link A contrast) revealed electrode activation in the delta and theta bands (Figure 7). In the delta band, a slightly left lateralized pattern of negative BSRs was observed in the frontal and temporal regions, also including one central posterior electrode. In the theta band, the effect of parts was localized in one medial posterior and one medial occipital electrode.

Third and finally, task PLS analysis of the Non-link B vs. Non-link A conditions failed to detect any electrode activation after FDR correction and thresholding.

## Discussion

Enabled by the digitizing tablet technology, this is the first study to characterize the intra-test behavior and associated EEG findings for TMT performance in young healthy adults. The study findings support the hypotheses, demonstrating a significant effect of time periods (non-linking, linking); and of TMT part (A, B) during TMT performance – as quantified by tablet-based kinematic metrics and EEG time-frequency power. The spatial dependence of the EEG findings is consistent with the existing fMRI and EEG literature investigating the TMT and reporting differences in brain activity between parts A and B. The overall findings are subsequently discussed in detail below, focusing first on behavioral results obtained from tablet-based kinematic recordings, and then on the EEG results. Limitations of the study are also indicated, as these have a potential influence on how the results are interpreted.

### Behavior

As an extension from previous investigations of the digitized TMT (Lara-Garduno et al., 2016, 2019; Dahmen et al., 2017), the present work demonstrated the utility of the state-of-the-art tablet system by characterizing behavioral dynamics within TMT and between TMT parts, as well as establishing a preliminary behavior-EEG relation that guided offline EEG data analysis for the first time. Supporting the study hypotheses, the effect of TMT part (TMT-B versus TMT-A) was evident in both digitized tablet metrics (SPL, linking and non-linking periods) and conventional metrics (completion times, number of errors).

As an initial study to prepare for future EEG-fMRI research, a block design TMT experiment with fixed block duration was adopted in the present work. Therefore, it was necessary to account for the fact that not all of the participants completed the TMT parts within the chosen block duration. The seconds per link metric SPL was adopted here, and was previously developed to assess TMT performance for each participant based on their full or partial performance of the test in a block design experiment (Karimpoor et al., 2017). Excluding the first trials, 87.5% of the TMT-A trials were completed within the 40 s block duration, whereas 97.3% of the TMT-B trials were completed within the 60 s block duration. For the incomplete trials, the median total number of links was 21 (range: 10-23) in TMT-A, whereas the median was 19 (range: 16-23) in TMT-B. In such cases, given that errors in TMT performance were negligible, the completion time can be estimated by multiplying the SPL value by the correct number of links to be performed in each part (24). Significant correlations have been previously shown between tablet and paper SPL values; although with tablet SPLs shown to be larger than paper SPLs (Karimpoor et al., 2017; Talwar et al., 2020). Such results indicate that tablet SPL values are a reasonable approximation of paper TMT performance, but that inherent differences are still evident between tablet- and paper-based test administrations: in environment, posture, sensation and subsequent motor performance.

In the present study, SPL values for TMT-B were significantly larger than the values for TMT-A, consistent with previous behavioral results (Karimpoor et al., 2017; Talwar et al., 2020). This supports the elevated cognitive demand of TMT-B compared to TMT-A. In addition, the distribution of SPL values was also evaluated by binning the SPL values for each trial for all participants. The distributions were both found to be right-skewed, indicating that participants took extended time to perform some of the trials in TMT-A and TMT-B, compared to most of the trials. The distributions were found to be statistically different, in keeping with the effect of TMT part on SPL values, although no difference in skewness was found for the sample of participants studied. It is possible that a difference in skewness could be found if a study with a larger sample of young healthy adults was undertaken, as there was a substantial difference in the histogram frequency value for both parts in the bin range from approximately 0.7-1.0 s, corresponding to the fraction of trials which were conducted the fastest. For these trials, the median and 95% confidence intervals for part A and part B did not overlap – with TMT-A exhibiting the higher frequency, again consistent with the increased cognitive demand occurring in TMT-B.

The digitizing tablet also permitted a more detailed study of within-test behavioral performance of the TMT. Linking periods indicate purposeful stylus movements to connect two stimuli; and non-linking periods reflect visual search and cognitive processes as required to form motor plans prior to purposeful movement.

Both the non-linking and linking periods were found to be longer in TMT-B than TMT-A. Taken together with the SPL findings, these results indicate the elevated processing demands of TMT-B compared to TMT-A as longer time was required to search, plan, and execute individual links. In terms of average link speed, no significant difference was found between TMT parts, despite that the links in TMT-A were performed slightly faster than those in TMT-B. This is an interesting result, suggesting that participants performed intentional movements quite similarly in the two TMT parts – i.e., once they identified which stimuli were to be linked, the linking movement was performed without much influence from whether it was number-number linking or number-letter linking. It is also notable that the effect of TMT part was larger for the linking period than for the non-linking period, suggesting that the difference in cognitive challenge between the two TMT parts was pronounced in this time interval for the participants studied. In other words, higher cognitive processes were found to engage more during linking movement execution than preparation.

It was also found that the distributions for average link speed, non-linking period and linking period values across the participants were different when comparing performance of TMT-A and TMT-B, consistent with the interpretations given above regarding the effect of TMT part on the median values of each metric. A simulated “perfect responder” (performing TMT-A and TMT-B correctly with no cognitive effort and constant linking speed) produced highly similar metric distributions between TMT parts. Therefore, the observed differences between TMT parts in the metric distributions can be attributed to the differences in cognitive demand, as intended by the task design, and not differences in the spatial arrangement of the stimuli.

In summary, the digitizing tablet has enabled the characterization of multiple between- and within-test metrics of TMT performance (SPL, average link speed, non-linking period, and linking period) that provide more insight than the traditional method of scoring the test using completion time.

These results are specific to the population studied: young healthy adults. It may be that one of the metrics, or another not yet derived from the tablet data, is particularly sensitive and specific for characterizing patients with deficits in certain aspects of brain function. For instance, patients with traumatic brain injuries may experience difficulties in sustained attention, and set switching, leading to elevated non-link periods and larger effect size between TMT parts for this measure. Supporting the use of tablet-derived behavioral metrics, another commonly used NPT - the Clock Drawing Test - has been shown to have improved diagnostic accuracy and detection sensitivity in patients with early dementia and mild cognitive impairment when implemented on a tablet, and quantified using a “time-in-air” metric (defined as the stylus transition time from completing one stroke to starting the next without contacting the tablet surface) (Müller et al., 2017). Tablet-based NPTs are also amenable in principle to a data-driven approach to discriminate populations with different brain health characteristics, rather than using the *a priori* definition of specific kinematic variables.

### Brain Activity

During linking periods of TMT-A and TMT-B, participants moved their stylus rapidly on the tablet, which required focus on coordinating and executing the motor movements to link items. During non-linking periods, the stylus was held stationary or was moved slowly, suggesting the involvement of multiple cognitive processes in preparation for subsequent links, such as visual search, motor planning, working memory and set-switching. Identifying the non-linking and linking periods was important for improved characterization of TMT behavioral performance, and the same was true for interpreting the associated EEG signals. The effect of time period was sufficiently strong that an analysis without considering this effect would have led to misleading results. The use of task PLS analysis critically enabled the effects in the EEG recordings to be interpreted with respect to time period, TMT part, and electrode.

The omnibus PLS lead to a ranking of effects (from largest to smallest: time period; TMT part during linking period; TMT part during non-linking period) that motivated subsequent two-condition PLS analyses for more revealing evaluation of the patterns of activity across EEG electrodes, than was obtainable from the omnibus analysis alone. In support of the study hypotheses, the two-condition PLS analyses re-affirmed the effect of time period (linking period versus non-linking period) and TMT part (TMT-B versus TMT-A), but indicated that the latter was only significant during the linking period. Despite the arbitrary sign of the condition mean loadings in two-condition PLS analyses, increased negative signal strength (i.e., highly negative BSR) is interpreted as decreased frequency band power (i.e., greater desynchronization). Event-related desynchronization in EEG signals has been associated with higher cognitive processes (such as memory), sensory processing, and movement (Pfurtscheller and Lopes da Silva, 1999). Consistent with these observations, linking periods showed greater desynchronization than the non-linking periods, and TMT-B exhibited greater desynchronization compared to TMT-A during linking periods. It is likely that the balance between lower and higher cognitive processes is different in the two periods. These specific contrasts are subsequently discussed below in relation to the spectral power and spatial characteristics of the associated EEG signals.

As shown in Figure 5C, both link vs. non-link contrasts had larger mean loadings than the two TMT-B vs. TMT-A contrasts. The effect of time period demonstrated different electrode spatial patterns in all five frequency bands examined: delta, theta, alpha, beta, and gamma. Compared to linking periods of both parts, non-linking periods exhibited a widespread increase in delta band power, with the exceptions of a few frontal and occipital electrodes. Delta oscillations have long been implicated in attention processes (Knyazev, 2012), especially as the mechanism underpinning selective attention (Lakatos et al., 2008). Increased delta power in the resting-state is also associated with increasing activity in the default mode network (DMN) (Meerwijk et al., 2015). Therefore, the results suggest varying attentional demands and distinctively engaged functional networks during visual search and focused motor action. The sources underlying the detected delta band power remain to be directly determined. Based on previous simultaneous EEG and fMRI research (Dang-Vu et al., 2008), the difference in delta activity may be originated from the precuneus, posterior cingulate, inferior frontal cortex, and medial prefrontal cortex, which were also shown to be involved during TMT performance in previous fMRI studies (Karimpoor et al., 2017; Talwar et al., 2020).

With regards to theta band activity, non-linking periods demonstrated uniformly increased theta band power across the scalp, when compared to linking periods. Commonly found in the hippocampus (Sirota et al., 2008), sensory cortex (Raghavachari et al., 2006), and cingulate cortex (Onton et al., 2005), theta activity has been associated with working memory (Gevins et al., 1997; Klimesch, 1999; Vertes, 2005; Raghavachari et al., 2006; Mitchell et al., 2008) and long-range synchronization (von Stein et al., 2000). Some EEG-fMRI research supports the association between the hippocampus, the cingulate and theta activity (Sammer et al., 2007), but some does not (Laufs et al., 2003b). An increase in theta activity has also been found to be negative correlated with fMRI brain activity in regions that overlap the DMN (Scheeringa et al., 2009), indicating heightened alertness (Tyvaert et al., 2008), and theta and gamma waves have been found to be phased-coupled throughout the cortex (Canolty et al., 2006) especially during visual working memory tasks (Schack et al., 2002). Therefore, the widespread increased theta power in non-linking periods is suggestive of a globally heightened alert state during visual search and motor planning, prior to motor execution.

Compared to linking periods, a widespread increase in alpha activity was evident in non-linking periods, especially in bilateral frontal regions. Originating from cortical and thalamic sources (Lopes da Silva et al., 1980; Lopes da Silva, 1991; de Munck et al., 2007), alpha activity exhibits negative fMRI signal correlations in cortical and DMN regions, and positive correlations in the thalamus (Laufs et al., 2003a, 2006; Gonçalves et al., 2006; de Munck et al., 2007; Tyvaert et al., 2008). Prefrontal alpha synchronization is indicative of internally oriented cognitive processing and attention, which is typically seen during mental imagery and planning (von Stein and Sarnthein, 2000; Knyazev, 2007), whereas decreased occipital alpha synchronization is associated with externally oriented attention (Cooper et al., 2003) and visual perception (Klimesch et al., 1998). Alpha band power is also implicated in memory (Klimesch, 1999, 2012), and negatively correlated to fMRI signals in deep and superficial layers of the visual cortex (Scheeringa et al., 2016). Synthesizing these findings from the literature, therefore, increased alpha band power in non-linking periods likely reflects the internally oriented cognitive processing triggered by visual perception of (and visual attention to) the test stimuli. Working memory during TMT performance may also contribute to the increased alpha band power. Given the widespread spatial pattern with a frontal projection, the detected difference in alpha activity may be the product of frontal cortical and thalamic generators as well as their interactions. This is somewhat consistent with fMRI TMT results, with the caveat that thalamic activity was not observed strongly and required exploratory analysis with a relaxed cluster-size threshold (Karimpoor et al., 2017; Talwar et al., 2020). In addition, such results also suggest that mu rhythms (the alpha waves found in motor-related areas) were in turn suppressed during linking periods, consistent with previous EEG research (Muthukumaraswamy et al., 2004; Pfurtscheller et al., 2006).

Compared to linking periods, non-linking periods showed increased and slightly right-lateralized beta band power in several frontal and prefrontal electrodes. A previous study has reported increased beta power immediately after stimulus onset, which is then followed by a power decrease during movement preparation and execution (Zaepffel et al., 2013). Beta waves originating from the deep layers of prefrontal cortex are associated with working memory encoding, retention, retrieval, and reallocation (Bastos et al., 2018; Lundqvist et al., 2018; Miller et al., 2018); whereas beta waves generated by the primary motor cortex during motor activity play a role in associating sensory input with the motor command (Hari and Salmelin, 1997; Jackson et al., 2002; Baker, 2007). In the present context, the increased beta band power in non-linking periods is consistent with more involvement of working memory (and not sensorimotor processes) during which the participants were actively engaged in visual search and forming motor plans to link stimuli with specific numeric and alphabetic relationships. The right prefrontal lateralization may be related to TMT-B sequencing and shifting errors, as previously shown to be associated with lesions in the right hemispheric dorsolateral prefrontal cortex (Kopp et al., 2015). This suggests that the right lateralization may be important to maintain performance accuracy in TMT. Furthermore, a simultaneous EEG-fMRI study identified a positive correlation between beta power and fMRI signals in the posterior cingulate, precuneus, and prefrontal cortex (Laufs et al., 2003b). In the present study, only the latter region showed elevated beta power in non-linking periods compared to linking periods, likely because the posterior cingulate and precuneus overlap with the DMN (Raichle et al., 2001) and are generally suppressed during fMRI studies of the TMT (Karimpoor et al., 2017; Talwar et al., 2020). The beta power results of Figure 6 also suggest that the DMN is similarly suppressed during non-linking and linking periods of TMT performance.

Compared to linking periods, non-linking periods showed a slightly right lateralized gamma band power increase in a few prefrontal electrodes. In animal studies, gamma waves have been associated with sensory and complex cognitive processes such as perception (Gray et al., 1989), attention (Fries et al., 2001), and memory (Tallon-Baudry et al., 1998). The sources of gamma waves have yet to be identified in human brain but may be located in the somatosensory cortex and primary visual cortex, inferring from animal studies (Chrobak and Buzsáki, 1998; van Kerkoerle et al., 2014). Therefore, a high neuronal excitability (i.e., increased sensitivity to synaptic inputs) during visual search and cognitive processes such as memory and set-switching is probable. In addition, the decrease in right lateralized prefrontal gamma activity in linking periods might represent a mechanism to inhibit neural activity that interferes with linking-related sensorimotor and cognitive processes (Ishii et al., 2014). It is also important to note the ongoing debate around the functional roles of gamma waves. Some studies report that gamma waves are associated with feature binding mechanisms (Von der Malsburg and Schneider, 1986; Singer, 1999) and intrinsic network properties (Steriade and Amzica, 1996; Steriade et al., 1996), whereas others report that they originate simply from oculomotor (Yuval-Greenberg et al., 2008) and muscle artifacts (Whitham et al., 2007, 2008). Further source estimates and topographic analyses are required to identify sources of gamma oscillations while excluding non-neuronal electrical activity in the EEG signal. In addition, it is interesting that the gamma and strongest alpha signals are largely from the same brain regions; therefore, improved SNR and data quality of EEG are needed to examine phase amplitude coupling between gamma and alpha signals in the future TMT studies.

Regarding the effect of TMT parts in linking periods, TMT-A demonstrated increased delta power with a modest left lateralization compared to TMT-B in three left temporal electrodes, two right frontal electrodes, and one electrode near the primary motor cortex. Such left lateralization suggests the elevated executive demand in TMT-B performance, which was also shown in the previous TMT studies of brain activity (Lorig et al., 1986; Stuss et al., 2001; Karimpoor et al., 2017; Talwar et al., 2020). However, no effect of part was detected in non-linking periods. The EEG data are consistent with the interpretation of less attentional demand and slightly more DMN activity in TMT-A than in TMT-B. During TMT performance, attention plays an important role in both linking and non-linking periods, and notably the left lateralization was only detected in the linking periods rather than the non-linking periods. A plausible interpretation of these findings is as follows: less cognitive effort is required to execute motor actions in linking periods of TMT-A; whereas in those of TMT-B, the higher cognitive processes such as set-switching and working memory remain engaged to maintain both letter and number sets. Alternatively, analogous visual search and motor planning components may dominate the EEG signal in non-linking periods of both TMT parts, thus diluting the signal differences due to executive functioning.

The EEG signals for TMT-A performance demonstrated increased theta band power in central posterior and occipital regions compared to those for TMT-B. Such results support the interpretation that TMT-A is associated with decreased working memory demand in relation to TMT-B. Given the involvement of working memory in the TMT (Oosterman et al., 2010) and previous fMRI findings (Karimpoor et al., 2017; Talwar et al., 2020), the detected difference in theta waves is likely to originate from sources such as the hippocampus, sensory cortex, and cingulate cortex, as theta waves from all three regions play a role in working memory.

It is premature to draw direct comparisons between the EEG findings of the present study and brain regions identified at approximately 1 mm spatial resolution in fMRI studies of TMT performance. Nevertheless, preliminary speculations can be made based solely on the visual similarities on the signal distribution patterns between the two modalities. It is interesting that the EEG spatial saliences are visually consistent with what would be expected given the fMRI literature. Specifically, the delta power increase was observed in the proximity of the right SMA (medial frontal gyrus), right premotor cortex, left inferior frontal gyrus, and left precuneus, suggesting that TMT-B performance requires more executive functioning and motor-related processing than TMT-A. This result is consistent with previous simultaneous EEG-fMRI research showing association between BOLD signals and delta band activity in the right medial frontal gyrus and left inferior frontal gyrus (Dang-Vu et al., 2008). The theta power increase was found near the precuneus, posterior cingulate cortex, and visual cortex, reflecting an increased memory demand and suppression of DMN during TMT-B performance compared to TMT-A. This interpretation is supported by past simultaneous EEG-fMRI experiments showing the relation between theta band activity and the cingulate cortex (Sammer et al., 2007; Scheeringa et al., 2009). These regions have all been shown to be involved during TMT performance in previous fMRI studies (Karimpoor et al., 2017; Talwar et al., 2020), but a direct relationship between the results of EEG and fMRI is not presently supported as EEG source localization and quantitative cross-modality comparison have not been conducted. The present study provides initial supportive data that sets the stage for future simultaneous EEG-fMRI studies, in which the two signal modalities will be recorded concurrently and compared quantitatively in the space and time domains.

It is interesting that the EEG results during linking periods show most agreement with the previous fMRI literature. The effect of time period is very strong in the EEG results, in keeping with the biophysical mechanism of signal contrast and postulated neural processing, whereas this effect has not previously been investigated in TMT-related fMRI research. As the linking period is longer than the non-linking period, it probably predominates the fMRI results that have been reported to date. However, future research could be undertaken using very high temporal resolution fMRI (Lin et al., 2008, 2014) to study differences in hemodynamic responses in the non-linking and linking periods.

The present EEG results somewhat agree with the past EEG study of the TMT (Lorig et al., 1986). In terms of high frequency oscillations in both TMT parts, beta and gamma band power were found to be more active in the frontal regions than posterior regions of the brain when comparing time periods, which is consistent with the past EEG study. No significant difference between TMT parts was found in beta and gamma band power, which somewhat agrees with the similar spatial patterns between parts observed in the past EEG study. A more left lateralized posterior brain activity in TMT-B than TMT-A was suggested in the past EEG study, but was not detected here in the effect of TMT part. In the present work, the effects of time period and part were evaluated, and spatial dependence of the EEG signals was studied at the electrode level with a denser electrode array and much more sophisticated multivariate analysis informed by tablet-based kinematic metrics. Due to the differences in test design, experimental hardware, and data analysis techniques, many EEG results were not directly comparable between the past EEG study and the present study.

There are a few limitations to the present study. First, the TMT is administered only once in the clinic to assess cognition; however, the EEG data analysis required multiple TMT trials of self-consistent data to provide sufficient signal-to-noise ratio (SNR), inevitably introducing practice effects. This was subsequently confirmed in preliminary statistical analysis showing that participants performed the first trial of TMT-A and TMT-B more slowly than the other trials. In the future, advanced functional neuroimaging techniques should be developed to extract meaningful interpretation from a single TMT trial for enhanced clinical relevance. Although this may be difficult to do with EEG, it may be possible to achieve with fMRI conducted at ultra-high fields, such as 7 T and above. Second, because the cognitive processing was asynchronous, complex, and continually varying during TMT performance, and across participants, it was challenging to classify and discriminate the linking and non-linking periods with complete certainty while ensuring that participants were not pursuing other strategies (such as searching for the next stimuli to be linked while performing the current link). Future studies combining EEG recordings with eye-tracking would be useful to characterize the visual search performed by participants during TMT performance, enabling periods with consistent behavior to be better identified. An alternative is to adapt the TMT paradigm for EEG into separate individual cognitive components, similar to the Delis-Kaplan Executive Function System (D-KEFS) TMT, a popular TMT variant widely used clinically (Delis et al., 2001). Third, the modest sample size and narrow participant demographics of the present study placed a limit on the effect sizes that could be detected with statistical significance, and introduced uncertainty about whether the results are fully representative of a larger population of young healthy adults. Conducting an additional EEG study of TMT performance in a larger sample size with more diverse demographics would be useful to investigate whether the present initial findings can be replicated, and whether smaller yet relevant effects are detectable, before proceeding to investigate patient populations. Fourth, the study did not include a comparison between paper- and tablet-based versions of the TMT to assess ecological validity of the behavioral and EEG findings. Previous comparisons of paper- and tablet-based TMT performance are available in the literature for fMRI settings in which participants used the tablet lying down, reporting good ecological validity (Karimpoor et al., 2017; Talwar et al., 2020). As participants interacted with the tablet while in a sitting posture for the present study, ecological validity is expected to be improved over what was reported in these previous studies – although this remains speculation. Fifth, the tablet analysis approaches such as video inspection and speed threshold analysis are preliminary. There was only one rater who watched the TMT video files to enable tablet data analysis, and thus no inter-rater reliability statistics have been reported. A fixed speed threshold for each trial is also suboptimal at extracting the precise timing of linking and non-linking periods, because of performance variabilities in linking behavior. In the future, these analyses can be improved and automated to deliver results more rigorously. Regarding such improved analyses, it is also notable that an in-depth error analysis was not conducted in the present study, because the cohort of participants (young healthy adults) committed negligible errors. An automated analysis of errors in various categories, such as TMT-B shifting and sequencing errors, will be required for subsequent research involving patients. Sixth and last, the present study used a modern but relatively simple 32-channel EEG system, and enhanced EEG technology could be used to enhance SNR in future studies. For example, higher electrode count, in conjunction with electromyography and electrooculography would be useful to identify and suppress non-neuronal electrical signal artifacts. Simultaneous fMRI-EEG studies would also be useful to assist in source localization and direct characterization of the relation between neural oscillations and fMRI signals in detailed neuroanatomical structures, advancing beyond the EEG electrode analysis reported here.

## Conclusion

In conclusion, longer SPL, linking and non-linking periods are evident in TMT-B compared to TMT-A, reflecting the increased cognitive demand in TMT-B, whereas link speed only exhibited such effect when comparing distributions, indicating a modest but dynamic sensitivity to cognitive processing. Regarding brain activity, TMT-A exhibited increased left lateralized delta band power and posterior midline theta band power compared to TMT-B, indicating less attentional and working memory demands in TMT-A, as expected. Non-linking periods demonstrated widespread increases in slow oscillatory activities in the delta, theta and alpha bands, suggesting a decreased attentional demand when the stylus moved slowly or was stationary, as well as a heightened alert state for increased internal processing to prepare for subsequent linking behaviors. In terms of fast neural oscillations, non-linking periods showed increases in beta and gamma band power in frontal regions with a slight right lateralization, reflecting working memory involvement, performance accuracy maintenance, and interference inhibition. Taken together, these effects in TMT part and within-test time periods support the study hypotheses. These observations contribute to increased understanding of the neural activity associated with TMT performance. As one of the few devices that permits simultaneous fMRI-EEG experiments, the digitizing tablet employed in the present study demonstrated utility by establishing the relationship between EEG and tablet data that informs future design of multi-modal functional neuroimaging experiments and multivariate data analysis.

## Data Availability Statement

The raw data supporting the conclusions of this article will be made available by the authors, without undue reservation.

## Ethics Statement

The studies involving human participants were reviewed and approved by the Research Ethics Board of Sunnybrook Health Sciences Centre. The patients/participants provided their written informed consent to participate in this study.

## Author Contributions

ZL, SG, and TS contributed to conceptualization of the study. ZL and SG contributed to investigation, data curation, and visualization. FT, NC, and SG provided the resources. ZL performed the formal analysis and wrote the first draft of the manuscript. ZL, FT, NC, and SG established the methodology. ZL and FT prepared the software. FT and SG validated the experimental results. SG acquired the funding and managed the project. F-HL, BM, TS, and SG supervised the study. All authors contributed to manuscript revision, read, and approved the submitted version.

## Conflict of Interest

The authors declare that the research was conducted in the absence of any commercial or financial relationships that could be construed as a potential conflict of interest.
